# Lipid Nanoparticle Technology for Delivering Biologically Active Fatty Acids and Monoglycerides

**DOI:** 10.3390/ijms22189664

**Published:** 2021-09-07

**Authors:** Jia Ying Brenda Tan, Bo Kyeong Yoon, Nam-Joon Cho, Jasmina Lovrić, Mario Jug, Joshua A. Jackman

**Affiliations:** 1 School of Chemical Engineering and Biomedical Institute for Convergence at SKKU (BICS), Sungkyunkwan University, Suwon 16419, Korea; btan061@e.ntu.edu.sg (J.Y.B.T.); bky0622@skku.edu (B.K.Y.); 2 School of Materials Science and Engineering, Nanyang Technological University, Singapore 637553, Singapore; njcho@ntu.edu.sg; 3 School of Healthcare and Biomedical Engineering, Chonnam National University, Yeosu 59626, Korea; 4 Department of Pharmaceutical Technology, Faculty of Pharmacy and Biochemistry, University of Zagreb, 10000 Zagreb, Croatia; jlovric@pharma.hr (J.L.); mjug@pharma.hr (M.J.)

**Keywords:** fatty acid, monoglyceride, lipid nanoparticle, liposome, bicelle, solid lipid nanoparticle, nanostructured lipid carrier, nanoemulsion, lipid nanocapsule, polymeric micelle

## Abstract

There is enormous interest in utilizing biologically active fatty acids and monoglycerides to treat phospholipid membrane-related medical diseases, especially with the global health importance of membrane-enveloped viruses and bacteria. However, it is difficult to practically deliver lipophilic fatty acids and monoglycerides for therapeutic applications, which has led to the emergence of lipid nanoparticle platforms that support molecular encapsulation and functional presentation. Herein, we introduce various classes of lipid nanoparticle technology and critically examine the latest progress in utilizing lipid nanoparticles to deliver fatty acids and monoglycerides in order to treat medical diseases related to infectious pathogens, cancer, and inflammation. Particular emphasis is placed on understanding how nanoparticle structure is related to biological function in terms of mechanism, potency, selectivity, and targeting. We also discuss translational opportunities and regulatory needs for utilizing lipid nanoparticles to deliver fatty acids and monoglycerides, including unmet clinical opportunities.

## 1. Introduction

Phospholipid membranes play integral roles in a number of medical diseases related to infectious pathogens [1,2], cancer [3,4], and inflammation [5,6] among various conditions. Global health challenges such as membrane-enveloped viruses with pandemic potential [7] and the growing number of antibiotic-resistant bacteria [8] have heightened general interest in developing therapeutic strategies to modulate and/or disturb lipid membranes surrounding cells and biological nanoparticles such as virus particles and exosomes [9,10]. Among various molecular drug options that interact with phospholipid membranes, naturally occurring fatty acids and monoglycerides have received extensive attention due to a wide range of molecule-specific biological activities, natural abundance, low cost, and perceived safety for dietary consumption and medical use [11].

**Figure 1:**
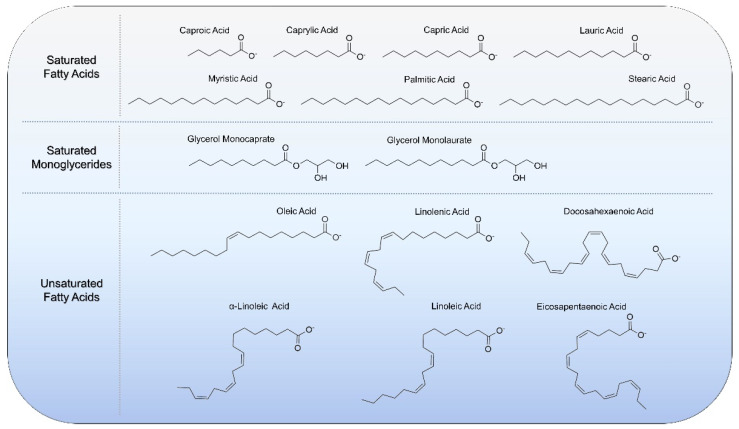
Molecular structures of biologically active fatty acids and monoglycerides that have been utilized with lipid nanoparticle technologies.

Fatty acids and monoglycerides are single-chain lipid amphiphiles that possess a hydrocarbon chain and a hydrophilic headgroup, and can intercalate into phospholipid membranes to trigger membrane disruptions such as solubilization, pore formation, or shape remodeling depending on the context [12]. Such membrane-modulating activities have proven useful for exploring fatty acids and monoglycerides, especially those with 6- to 12-carbon long, saturated chains, as anti-infective agents to inhibit membrane-enveloped viruses and bacteria [13,14], along with longer-chain, polyunsaturated fatty acids for cardiovascular and immune health [15]. An overview of the molecular structures of biologically active fatty acids and monoglycerides is presented in Figure 1 and there are various classes depending on the headgroup properties, chain length, and prevalence and number of double bonds in the chain.

However, despite the useful biological activities of fatty acids and monoglycerides, critical hurdles to translational applications must be overcome. Indeed, fatty acids and monoglycerides are rather poorly soluble and require supramolecular organization to be biologically active in many cases. Hence, practically realizing therapeutic delivery of fatty acids and monoglycerides calls for the development of effective pharmacological strategies to encapsulate fatty acids and monoglycerides in configurations that not only support high loading capacity but also permit retention, or even enhancement, of membrane-modulating biological activities. These design requirements have led to the development of numerous, increasingly sophisticated nano-carriers to encapsulate and deliver fatty acids and monoglycerides [16,17], a topic of nanomedicine that is experiencing a renaissance in general due to the widespread implementation of lipid nanoparticle technology for vaccine delivery applications [18] and the broader translational possibilities that such developments inspire.

Herein, we cover the latest progress in developing lipid nanoparticle technology to deliver biologically active fatty acids and monoglycerides across applications spanning infectious diseases, cancer, and inflammation as well as for small-molecule drug delivery enhancement. We first introduce the basic design principles of various lipid nanoparticle technologies and related nano-carriers involving other lipid-like building blocks, all of which have been used to encapsulate fatty acids and monoglycerides, and then provide application examples. Lastly, we critically discuss some of the biggest needs and unmet opportunities in the field, including areas where we see high translational potential and possible steps to reach these goals.

## 2. Why Nano?

While fatty acids and monoglycerides can have important biological activities, it is challenging to utilize these molecules as therapeutic drugs because they are mainly active in supramolecular structures. Practically, this means they are only active at sufficiently high concentrations, at which they begin to self-assemble into micellar nanostructures. However, such micelles readily lose structure upon dilution and fatty acid and monoglyceride monomers are mainly inactive. The translation of fatty acids and monoglycerides into clinically feasible therapies is further challenged by formulation hurdles caused by low aqueous solubility and dispersibility [16,19]. To address these issues, there has been extensive attention placed on developing lipid nanoparticle technologies to encapsulate biologically active fatty acids and monoglycerides and there are several compelling reasons to do so: (1) stable supramolecular structures enable biological functionality of fatty acids and monoglycerides along with improving dispersibility; (2) nanoscale size is ideal for interfacing with biological targets such as bacterial cells and virus particles; and (3) additionally, nanoscale size enables the potential for cellular uptake, which could lead to fatty acids and monoglycerides exhibiting both intracellular and extracellular activities.

Moreover, lipid nanoparticles are the most common class of FDA- and EMA-approved nanomedicines [20], representing a technological platform that offers an assortment of modifiable features such as size, shape, charge, surface properties (including ligand presentation), and responsiveness that can be engineered to increase cargo loading capacity, chemical stability, and capability to cross various biological barriers depending on the application context. The fundamental concepts, mechanisms, and emerging strategies used in nanoparticle development to overcome biological barriers on the systemic, local, and cellular levels have been recently reviewed elsewhere [21,22,23,24,25,26].

**Figure 2:**
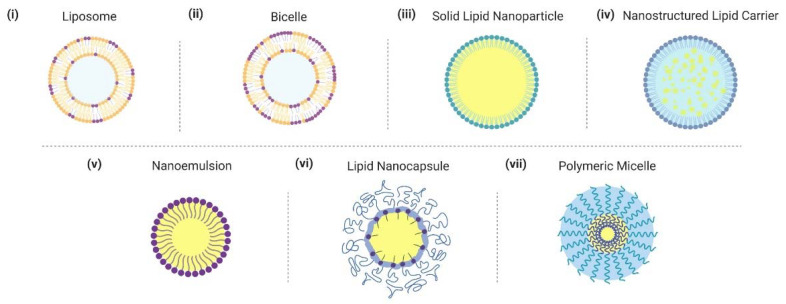
Overview of lipid nanoparticle strategies and related nano-carrier formulations for delivering fatty acids and monoglycerides. Graphical illustrations of different nanoparticles are presented. Nanoparticles are not drawn to scale.

In this section, we critically introduce different examples of lipid nanoparticle technologies that have been developed for delivering fatty acids and monoglycerides (Figure 2). Particular emphasis is placed on nanostructures where lipid molecules play critical roles in defining the architecture while additional nanostructures involving lipid-like, amphipathic building blocks such as oils and polymers are also discussed as applicable. While the basic design aspects of different lipid nanoparticle technologies have been described in past works, we emphasize specific features that can affect application performance in the present context.

### 2.1. Liposome

Liposomes are spherically enclosed lipid bilayers that can self-assemble in aqueous solution and have an aqueous core [27]. They typically have ~50–300 nm diameter and the mechanical properties can be adjusted depending on the application context. Gel-phase phospholipids and cholesterol-containing compositions tend to form mechanically strong liposomes while fluid-phase phospholipids form more flexible liposomes. While liposomes have been explored for delivering fatty acids and monoglycerides, they have relatively low loading capacities for these molecules in the liposomal bilayer.

### 2.2. Bicelle

Bicelles typically consist of a binary mixture of long-chain and short-chain phospholipids and can form a variety of morphologies [28]. Depending on the morphology, bicellar nanostructures usually have ~50–500 nm diameter and recent designs have focused on utilizing fatty acids and monoglycerides in place of short-chain phospholipids [29,30]. While the discoidal aggregate structure is commonly associated with bicelles [31], the vesicle-like morphology of bicellar nanostructures is useful for delivering fatty acids and monoglycerides. Such structures can be formed with high fractions of monoglycerides and exhibit membrane softening that is useful for enabling fusogenic activities [32].

### 2.3. Solid Lipid Nanoparticle

Solid lipid nanoparticles (SLNs) are ~50–300 nm diameter nanoparticles that can be produced using fully aqueous conditions and have relatively high loading capacity [33]. The core consists of lipids (often stearic acid) that are in the solid (gel) phase at ambient and body temperatures, and there is a surfactant coating that aids stability. Depending on the preparation method and formulation specifics, amphipathic molecules such as fatty acids and monoglycerides can be included in the surfactant layer and/or core. While SLNs have many advantageous properties, solidification and gradual crystallization of the core structure during storage can cause nanoparticle instability [33].

### 2.4. Nanostructured Lipid Carrier

As an improved version of SLNs, nanostructured lipid carriers (NLCs) possess a core that is composed of a mixture of solid (gel) and liquid (fluid) phase lipids [34]. The solid lipid typically consists of stearic acid or a related, long-chain monoglyceride and the liquid lipid is often oleic acid, a mixture of medium-chain mono-, di-, or triglycerides, or a natural oil. In general, NLCs can offer improved loading capacity while the specific NLC composition should be optimized depending on the particular drug compound.

### 2.5. Nanoemulsion

Oil-in-water nanoemulsions (NEs) have typical droplet sizes in the 20–200 nm diameter range and are stable kinetically [35]. They consist of an oily core and are surrounded by an outer layer of surfactants, which can include fatty acids and monoglycerides. Depending on the relative proportions of the aqueous phase, oil phase, and nonionic surfactant along with temperature cycling conditions, lipid nanocapsules (LNCs) can form instead of nanoemulsions [36] and are described below.

### 2.6. Lipid Nanocapsule

Lipid nanocapsules (LNCs) are ~50–100 nm diameter nanoparticles that are fabricated in solvent-free conditions by the phase inversion temperature method [37]. Structurally, LNCs consist of an oily core (often medium-chain triglycerides) that is typically surrounded by an outer shell of surfactant and lecithin. Additional surfactant-like molecules such as fatty acids and monoglycerides can also be included in LNC formulations.

### 2.7. Polymeric Micelle

Polymeric micelles are ~10–200 nm diameter nanostructures that self-assemble from amphipathic block copolymers in aqueous solution [38,39]. They have a hydrophobic core and hydrophilic shell and hydrophobic and/or amphipathic molecules are typically loaded into the core. The specific molecular properties of the polymeric micelle design is important because the nanostructure must be strong enough to remain stable upon dilution but also allow the release of encapsulated molecules [38].

## 3. Application Examples

In this section, we critically introduce application examples where nanostructured assemblies incorporating fatty acids and monoglycerides have demonstrated promising biological activities. In applicable cases, we discuss how the nanostructure assembly enables greater activity of the fatty acid or monoglyceride molecules compared to their free forms, e.g., by facilitating supramolecular organization of the fatty acids and monoglycerides at concentrations below the critical aggregation concentration of the corresponding molecules by themselves.

### 3.1. Infectious Diseases

One of the most active areas of investigation involves targeting infectious pathogens such as bacteria and viruses. For such applications, the membrane-disruptive properties of fatty acids and monoglycerides play an important role by inducing bacterial cell membrane disruption that causes cell death and/or inhibits viability and by abrogating the infectivity of membrane-enveloped virus particles through physical disruption. These inhibitory functions can be especially useful in situations where the pathogen load is high, and we present examples related to antibacterial and antiviral activities.

#### 3.1.1. Antibacterial

Among single-chain lipids, medium-chain fatty acids and monoglycerides with 6- to 12-carbon long hydrocarbon chains have been reported to demonstrate particularly high levels of antibacterial activity [40]. Moreover, a few studies have demonstrated that they have a high barrier to resistance development [41], which makes them promising antibiotic alternatives. However, as mentioned above, medium-chain fatty acids and monoglycerides are mainly active in micellar aggregate form and appreciably less active in monomeric form, which has limited therapeutic applications due to micellar collapse upon dilution in physiological settings. Hence, there are ongoing efforts to develop nanostructured assemblies to encapsulate these fatty acids and monoglycerides in order to increase potency and dilution stability by virtue of supramolecular organization. A summary of these efforts is presented in Table 1.

We begin by introducing lauric acid (LA), which is a 12-carbon long, saturated fatty acid that has been explored for treating bacterial skin infections. Nakatsuji et al. reported that LA can inhibit the growth of common skin bacteria such as *Propionibacterium acnes* (*P. acnes*), *Staphylococcus aureus* (*S. aureus*), and *Staphylococcus epidermis* (*S. epidermis*) [57]. Notably, LA had greater potency in vitro than benzoyl peroxide, which is a widely used anti-acne medication. Intradermal injection and topical application of LA were also effective at reducing the amount of *P. acnes* in an in vivo mouse ear model and also decreased ear swelling and inflammation. In the present context, it should be emphasized that LA by itself is poorly soluble in aqueous suspensions and 5% v/v dimethyl sulfoxide (DMSO) was utilized in the formulation for the in vitro and in vivo experiments. Another study compared the antibacterial activity of LA with that of capric acid (CA), which is a 10-carbon long, saturated fatty acid, and showed that both compounds can inhibit *P. acnes* in vitro and in vivo [58]. It was also noted that LA had superior antibacterial activity, which has led to the development of nano-carrier strategies in place of utilizing organic solvents such as DMSO.

Yang et al. reported the development of a liposomal formulation to encapsulate LA with improved antibacterial activity against *P. acnes* [42]. The authors first verified that LA inhibited *P. acnes* while two other fatty acids, palmitic acid (PA) and oleic acid (OA), which are 16-carbon long, saturated and 18-carbon long, monounsaturated fatty acids, respectively, did not inhibit the bacterium. The ~120-nm diameter liposomes were prepared by the extrusion method and contained varying amounts of fluid-phase egg phosphatidylcholine (Egg PC) lipids and LA along with cholesterol to reinforce the liposomal structure. The LA mass ratio was varied from 0% to 40% (equivalent to roughly 0–70 mol%) and more LA increased the negative surface charge of the fabricated liposomes, which provided evidence that LA was present in the liposomal bilayer. Importantly, liposomal LA was able to inhibit *P. acnes*, while it was noted that a critical amount of LA per liposome (as opposed to considering only the total LA concentration in the system) must be present for maximum inhibitory effect. Fluorescence resonance energy transfer (FRET) experiments provided additional evidence that the LA-loaded liposomes fuse with the *P. acnes* cell membrane, whereby LA transfer can occur and results in bacterial killing.

A follow-up study reported that liposomal LA could be injected intradermally or delivered topically in a cellulose gel to inhibit *P. acnes* in an in vivo mouse ear model and the topical gel formulation was also shown to not cause skin irritation [43]. Liposomes encapsulating LA alone and together with curcumin in a gel formulation have also been explored for inhibiting *P. acnes* and co-encapsulation of the two compounds yielded superior antibacterial and anti-inflammatory activities compared to liposomes loaded with curcumin only [44]. Considering these advantageous points of liposomal LA, the fabrication and physicochemical characterization of ~100-nm diameter, presumably gel-phase 1,2-dipalmitoyl-*sn*-glycero-3-phosphocholine (DPPC) liposomes encapsulating up to 50 mol% LA have been reported [59]. The LA-loaded DPPC liposomes tended to form lamellar-phase nanostructures, mainly unilamellar and multilamellar liposomes, while the ratio between charged and uncharged LA molecules depended on the solution pH (7.4 or 5.0) and influenced the packing properties of the liposomal bilayers. At both pH conditions, the liposomes exhibited minimal penetration in an in vitro pig ear skin model, supporting that they are useful for skincare applications.

Depending on the target bacterium, different fatty acids and monoglycerides can exhibit particularly high antibacterial activity levels, which has motivated the further development of liposomal formulations for other compounds besides LA. For example, liposomal OA has been reported to potentially inhibit methicillin-resistant *S. aureus* (MRSA), which is a leading cause of hospital-acquired infections [45]. FRET measurements indicated that OA-loaded liposomes could rapidly fuse with MRSA bacterial cell membranes and liposomal OA was judged to have an approximately 12-time lower minimum bactericidal concentration (MBC; defined as fatty acid concentration corresponding to 99.9% bacterial cell killing) compared to free OA. The greater potency of liposomal OA was attributed to “burst release” of OA molecules during the fusion process, and intradermally injected liposomal OA also inhibited MRSA viability and infection-related inflammation in a mouse skin model. When applied to mouse skin tissue sections, liposomal OA exhibited good biocompatibility and no epidermal cell apoptosis was observed.

In another clinically relevant example, liposomal formulations of linoleic acid (LLA), an 18-carbon long, di-unsaturated fatty acid, were developed to treat *Heliobacter pylori* (*H. pylori*) infection, which is a leading cause of gastric diseases such as stomach cancer [46]. Initial studies focused on the development of ~88-nm diameter liposomes that were composed of LLA, Egg PC, and cholesterol in a 1:5:1 mass ratio and the liposomal LLA fused with *H. pylori* cell membranes, which resulted in bacterial cell killing. Additional tests showed that liposomal LLA could kill both spiral and coccoid forms of *H. pylori* and induced gross morphological disruption. Moreover, liposomal LLA inhibited a wide range of *H. pylori* strains and no resistance development was observed within the 10-day test period, while high concentrations of free LLA caused resistance development starting from day 3. Mechanistic studies have also revealed that liposomal LLA treatment increases *H. pylori* membrane permeability in vitro and rapidly damages the bacterial cell membrane, as indicated by loss of cytoplasmic contents and separation of the outer and inner membranes [47].

In an in vivo follow-up study, ~106-nm diameter liposomes consisting of LLA, Egg PC, and cholesterol in a 3:6:1 mass ratio were fabricated and liposomal LLA had a slightly more potent MBC value to kill *H. pylori* than free LLA [48]. Importantly, orally administered liposomal LLA markedly inhibited *H. pylori* by over 99% in the stomachs of infected mice, whereas orally administered free LLA was inactive. While previous studies demonstrated that liposomal fatty acids are therapeutically useful in localized contexts involving intradermal or topical applications, this study provided the first evidence that liposomal fatty acids can be useful for systemic applications with oral delivery. The in vivo results also underscore the importance of the liposomal structure in enabling supramolecular organization of the LLA molecules even upon dilution in physiological settings, whereas the free LLA molecules are likely to lose micellar structure and become inactive monomers upon dilution. Of note, the in vivo antibacterial performance of liposomal LLA was superior to that of a triple antibiotic cocktail therapy tested in parallel and liposomal LLA also reduced production of proinflammatory cytokines and did not harm gastric tissues. Additional in vitro experiments further indicated that liposomal LLA inhibited bacterial cells at concentrations that were not cytotoxic to stomach cells and the liposomal formulation protected against the cytotoxic effects of free LLA. Another study showed that orally administered liposomal LLA caused only minor changes in the gastrointestinal microbiota of mice, whereas the triple antibiotic cocktail therapy caused appreciable changes, including increases in bacterial populations that are associated with various diseases [49].

In addition to encapsulating fatty acids in liposomal formulations, there has also been recent exploration of bicellar nanostructures to encapsulate monoglycerides. Sut et al. reported the fabrication of lipid bicelles composed of 1,2-dioleoyl-*sn*-glycero-3-phosphocholine (DOPC) phospholipid and glycerol monolaurate (GML), which exhibited membrane-disruptive activity that was distinct from that of free GML [50]. The specific ratio of DOPC and GML components influenced the type and extent of membrane-disruptive activity and particular focus was placed on pore-forming lipid bicelles composed of DOPC and GML in a 1:4 molar ratio. These bicelles self-assembled into liposome-like, lamellar-phase nanostructures with 100–600 nm diameters and exhibited antibacterial activity against *S. aureus*. It should be emphasized that the specific molecular properties of the bicelles affected the degree of antibacterial activity. For example, changing the molar ratio of DOPC and GML to 2:1 resulted in GML-loaded bicelles that did not exhibit antibacterial activity, and thus the nanoparticle design should be carefully considered.

Aside from membranous liposomal and bicellar nanostructures, there has been exploration of other types of nanostructure for encapsulating fatty acids and monoglycerides. Tran et al. also reported the development of polymeric micelles composed of poly(ε-caprolactone)-poly(ethylene glycol)-poly(ε-caprolactone) (PCL-PEG-PCL) micelles and LA in a 5:1 mass ratio [51]. Depending on the PCL-PEG-PCL molecular weight, the LA-loaded polymeric micelles had ~30–90 nm diameters and exhibited approximately two-fold more potent antibacterial activity against *P. acnes* than free LA alone. Solid lipid nanoparticles (SLNs) containing LA alone or together with OA have also been fabricated together with SA and the resulting nanoparticles were coated on tubing surfaces to inhibit *Pseudomonas aeruginosa* (*P. aeruginosa*) adhesion [52].

Another approach has been to fabricate lipid nanocapsules (NCs) that contain an oily core of medium-chain triglycerides and a surrounding layer of polyoxyl 15 hydroxy-stearate surfactant plus lecithin, fatty acid, or monoglyceride as a co-surfactant [53]. The tested fatty acids and monoglycerides included caproic acid (COA), caprylic acid (CYA), CA, LA, myristic acid (MA), palmitic acid (PA), and stearic acid (SA), which are 6-, 8-, 10-, 12-, 14-, 16-, and 18-carbon long, saturated fatty acids along with glycerol monocaprate (GMC; monoglyceride derivative of CA) and GML. The lipid NCs had sizes in the range of 40–90 nm diameter depending on the specific co-surfactant and all fatty acid- and monoglyceride-containing lipid nanoparticles inhibited *S. aureus* in vitro, whereas lecithin-containing lipid nanoparticles were inactive. Among the NC compositions, those containing fatty acids or monoglycerides with shorter chain lengths tended to have a wider range of inhibitory activity against Gram-positive and Gram-negative bacteria, while those containing fatty acids or monoglycerides with longer-chain lengths tended to inhibit Gram-positive bacteria more potently. It was noted that GML encapsulation in the NCs had the greatest protective effect to minimize hemolysis in vitro. GML-containing lipid NCs were further loaded with antimicrobial peptides via adsorption and demonstrated synergistic antibacterial activity against planktonic *S. aureus* [54]. A follow-up study investigated the antibacterial effects of GML-containing lipid nanocapsules with adsorbed antimicrobial peptides on *S. aureus* biofilms [55]. Reductions in biofilm growth were observed in vitro, and the GML-containing lipid nanoparticles supported quicker healing of infection-related wounds in a mouse model, but the peptide-NC combination did not display synergistic activity in vivo.

While the aforementioned studies focus on the antimicrobial properties of medium-chain fatty acids and monoglycerides, other types of biologically active lipids have also been explored for antibacterial applications, including docosahexaenoic acid (DHA), a 22-carbon long fatty acid with six degrees of unsaturation [56]. In particular, NLCs composed of glyceryl palmitostearate, medium-chain triglycerides, and Tween-60 surfactant were fabricated and loaded with varying amounts of DHA. The DHA-loaded NLCs had diameters around 300 nm and superior antibacterial activity to inhibit *H. pylori* compared to free DHA. In addition, the DHA-loaded NLCs caused extensive loss of cytoplasmic contents that was indicative of membrane disruption. In vitro experiments indicated that DHA-loaded NLCs affected the viability of stomach cells near the range of antibacterial concentrations, suggesting that further optimization is warranted to improve selectivity.

#### 3.1.2. Antiviral

The ongoing coronavirus disease 2019 (COVID-19) pandemic has caused a surge in interest in utilizing biologically active lipids to inhibit membrane-enveloped viruses such as severe acute respiratory syndrome coronavirus 2 (SARS-CoV-2) [60]. The antiviral properties of fatty acids and monoglycerides have long been known, but there are few studies discussing the antiviral properties of these compounds within nanostructured assemblies. To date, an emulsion mixture comprising lecithin, pluronic F-68 co-surfactant, and 1,2-dipalmitoyl-*sn*-glycero-3-phosphorylglycerol (DPPG) lipid along with caprylic acid and capric/caprylic triglyceride in the oil phase was developed to inhibit a broad spectrum of medically important, enveloped viruses [61]. The emulsions inhibited entry of virus pseudo-particles that presented the envelope glycoproteins of vesicular stomatitis virus, Lassa, Ebola, and severe acute respiratory syndrome coronavirus 1 (SARS-CoV-1) viruses, as well as infectious Zika virus particles. It was further identified that caprylic acid was the main antiviral component and caprylic acid within the emulsion had greater antiviral activity than free caprylic acid. On the other hand, the emulsion only inhibited membrane-enveloped viruses, but not non-enveloped viruses, which is consistent with the expected mechanism of action involving viral membrane disruption. Topical application of the emulsion formulation on top of mosquito bite sites prior to subcutaneous virus injection at the same site helped to reduce Semliki Forest and Zika virus replication in a mouse model. While this topic is an area of major opportunity, there remains an outstanding need to intensify research efforts, especially since membrane-disruptive antiviral peptides have demonstrated therapeutic efficacy in treating viral infections in vivo based on similar mechanistic concepts [62,63].

### 3.2. Cancer

In addition to medium-chain fatty acids and monoglycerides, there has also been interest in developing nano-carrier formulations to stabilize and deliver longer-chain fatty acids such as DHA and eicosapentaenoic acid (EPA) as well as other polyunsaturated fatty acids, which contain multiple double bonds in the hydrocarbon chains and are useful for preventing cardiovascular disease and inhibiting cancer cells. Various nano-carrier formulations have been explored, including liposomes that facilitate high loading of fatty acids into lipid bilayer environments [64]. While liposome-based nanomedicines are currently used to encapsulate small-molecule, chemotherapeutic drugs, the liposomes in those cases are strictly carriers and have reinforced membrane structures, including high cholesterol contents and polymer-modified lipids, in some cases, to enhance bioavailability [65]. On the other hand, the liposomes in the cases discussed below have a wider range of compositions that take into account the importance of the liposomal bilayer as not only a structural component, but also as an active interface. One of the most important applications involves anticancer therapy and a summary of these efforts is presented in Table 2.

Inspired by past work on liposomes containing fatty acid-enriched natural extracts [78], Tanaka et al. first reported the development of liposomes that contained 1000 μM dimyristoylphosphatidylcholine (DMPC) lipid and 111 μM Tween 80 surfactant along with 100 μg/mL DHA, EPA, LLA, OA, or α-linolenic acid (ALA) and ethyl ester derivatives [66]. The liposomes were fabricated using sonication and filtration and fatty acid-containing liposomes maintained consistent sizes in the ~50–200 nm diameter range ,while blank liposomes without fatty acid incorporation were more prone to aggregation, presumably due to the lack of colloidal repulsion (by virtue of anionic fatty acid incorporation). Compared to blank liposomes, the fatty acid-containing liposomes also inhibited a variety of cancer cell lines in vitro in most cases, except that the OA ethyl ester derivative was inactive against one cell line. It had been previously discussed how polyunsaturated fatty acids inhibit cancer cells via lipid peroxidation and can selectively kill human cancer cells at concentrations that do not adversely affect normal human or animal cells [79,80]. The same assays were also conducted in the presence of an antioxidant, which revealed that liposomes containing certain fatty acids or ethyl esters were inhibitory on account of peroxidation-related cytotoxicity while liposomes containing other fatty acids or ethyl esters also exhibited additional mechanisms of anticancer activity.

Among the different fatty acids, liposomal LLA and DHA exhibited particularly high levels of inhibitory activity and inhibited cancer cells via necrosis- and apoptosis-inducing mechanisms, respectively [67]. It was suggested that liposomal DHA deserved further attention for development on account of inducing cancer cell apoptosis. A follow-up study fabricated ~100-nm diameter DMPC liposomes containing 10–60 mol% DHA and maintained high colloidal stability over time [68]. With increasing DHA fraction, liposomal DHA also had decreased membrane fluidity and high levels of apoptotic DNA fragmentation were caused by liposomal DHA with 50 and 60 mol% DHA, while liposomal DHA with 10–40 mol% DHA caused lower fragmentation levels. Mechanistic analysis further revealed that liposomal DHA inhibits cancer cells by inducing apoptosis and differentiation. The liposomal DHA formulation with 50 mol% DHA was further investigated for in vivo therapeutic applications and intravenous administration of liposomal DHA improved treatment outcomes in a mouse model of liver cancer [69]. Improved outcomes included reduced liver weight (compared to tumor-bearing mice without treatment), decreased number of metastatic nodules, induction of cancer cell apoptosis within excised tumor tissue, and prolonged survival.

To facilitate clinical translation, Skibinski et al. developed an acid-stable version of liposomal DHA that consisted of DHA, 1,2-di-*O*-hexadecyl-*sn*-glycero-3-phosphatidylcholine, and 1,2-di-*O*-phytanyl-*sn*-glycero-3-phosphatidylethanolamine in a 1:6:3 ratio and the extruded liposomes had an ~140-nm diameter [70]. The liposomal DHA size remained stable across the tested range of pH conditions (1–7.4) and the liposomal formulation markedly protected DHA from oxidation, as compared to free DHA stored for 1 week at 4 or 37 °C. Compared to free DHA, liposomal DHA also exhibited greater human breast cancer cell inhibition in vitro and was more effective at inducing cancer cell apoptosis.

In addition to polyunsaturated fatty acids, OA also exhibits anticancer activity and a liposomal formulation composed of gel-phase DPPC lipid and cholesterol in a 7:3 molar ratio plus OA was prepared using the extrusion method [71]. The resulting liposomes had ~130-nm diameter, were physically stable, and had an OA encapsulation efficiency of around 51%. Liposomal OA exhibited potent cancer cell inhibition in vitro and a modest degree of selectivity over non-cancer cells, e.g., a certain liposomal amount strongly inhibited two cancer cell lines (<~15% viability) while normal (Vero) cell viability was maintained at ~60%. It was suggested that the degree of selectivity could be potentially improved by functionalizing the liposomal surface with tumour-homing molecules or by adjusting the OA amount per liposome.

Aside from liposomes, other nano-carriers have been explored for anticancer applications. For example, Mussi et al. developed ~80-nm diameter NLCs that contained DHA (in triglyceride form) and doxorubicin (DOX), which is a widely used chemotherapy drug, and maintained physical stability after incubation in serum [72]. The encapsulated DHA and DOX combination exhibited greater cancer cell inhibition in vitro than the free DHA and DOX combination, along with more extensive penetration in a tumor spheroid model. A follow-up study investigated the biodistribution of intravenously administered ~70-nm diameter NLCs containing DHA and DOX in tumor-bearing mice and noted a longer much longer circulation half-life of encapsulated DOX, as compared to free DOX [73]. The encapsulated DHA also had higher accumulation in the tumor region, supporting tumor accumulation of the NLCs. Importantly, the NLC encapsulating both DHA and DOX outperformed the equivalent nano-carrier with DOX only and free DOX to inhibit tumor growth and induce tumor tissue necrosis. Further incorporation of α-tocopherol succinate (TS)–a compound which has been proposed to increase the efficacy of anticancer chemotherapy drugs–within the NLC also led to potent tumor inhibition in a breast cancer mouse model [74]. It was noted that the NLC formulations caused similar effects to those of free DOX on most hematological and biochemical parameters, while additional evaluation is warranted.

Expanding on these developments, Lanna et al. fabricated various types of nanostructured assemblies, including NEs, NLCs, and polymeric NCs, that contained DHA (in triglyceride form) and artemether (ART), which is an antimalarial drug that has been explored as an anticancer therapy [75]. The nanostructures had ~150–200 nm diameters and co-loading with DHA and ART led to greater cancer cell inhibition in vitro than free DHA or ART only. The inhibitory activity of the nanostructures was moderately selective for cancer cells over normal cells (by ~4–7-fold for NCs and NEs, whereas NLCs had ~8-fold selectivity for one cancer cell line but no selectivity towards another cancer cell line). All three types could also be internalized by cancer cells, resulting in apoptosis. In addition, the incorporation of DHA and DOX in ~100-nm diameter SLNs has been explored and led to greater cancer cell inhibition in vitro, compared to free DHA and DOX [76]. Intracellular uptake of encapsulated drugs in the SLNs was also improved compared to that of the free drugs. Serini et al. have also reported the development of SLNs containing a lipidated version of resveratrol together with LNA or DHA in some formulations; the incorporation of either fatty acid led to improved cancer cell inhibition in vitro [77].

Interestingly, current studies in the field have mainly focused on looking at how lipid nanoparticles affect cancer cells in vitro and tumors in vivo, while there is an outstanding need to further understand how the reported treatment effects may relate to direct interactions with cancer cells, or are due to indirect effects on immune cells and other systemic components that impact the tumor microenvironment [81]. Further consideration of nanoparticle properties and surface functionalization may also improve tumor targeting properties by virtue of different mechanisms such as the enhanced permeability and retention (EPR) effect, transcytosis, and hitchhiking [82].

### 3.3. Inflammation

Various classes of fatty acids and monoglycerides have demonstrated anti-inflammatory effects by interacting with immune cells and other types of biological activities. For example, DHA is widely explored for immune support functions [83] while there are extensive efforts to improve lipid stability and biological function. As with other applications, liposomal formulations have been tested and there has been investigation of different liposome preparation methods. For example, Rasti et al. investigated the encapsulation of DHA and EPA in ~300–350 nm diameter liposomes and observed that a preparation method involving aqueous solvent only yielded greater oxidative stability compared to a preparation method that involved aqueous and organic solvents [84]. Further refinements of the liposomal formulation included α-tocopherol, which enhanced oxidative protection to increase stability [85,86].

Alaarg et al. developed DHA-containing, ~100-nm diameter DPPC liposomes that could be internalized by macrophage cells, which are involved in proinflammatory cytokine production along with generating reactive oxygen and nitrogen species [87]. The DHA-containing liposomes could be up-taken by, and inhibited, nitric oxide generation from activated macrophages by up to ~80%. They also significantly decreased nuclear factor kappa B (NF-κB) signaling and proinflammatory cytokine production. Moreover, the DHA-containing liposomes potently inhibited the proliferation of two cancer cell lines in vitro, but did not affect the proliferation potential of a non-cancer cell line (e.g., human endothelial cells). In addition to liposomal strategies and building on an SLN design with anticancer activity as described above (see ref. [77]), Serini et al. have also investigated the utility of DHA-containing, resveratrol-based SLNs to inhibit the effects of cytotoxic and pro-inflammatory antagonists on two skin keratinocyte cell lines [88]. The DHA-containing nanoparticles also protected against reactive oxygen species generation in the presence of hydrogen peroxide, and all the encapsulated DHA outperformed free DHA for all these protective functions. Thus, it was suggested that the DHA-containing nanoparticles can be useful to protect skin from environmental irritants.

A recent study also explored the development of oil-containing lipid NCs as an anti-inflammatory therapy and included antibody surface functionalization to target binding to atherosclerotic plaques that are related to an inflamed endothelium [89]. The oils consisted of DHA-enriched algae oil or medium-chain triglycerides containing mainly caprylic and capric acids. Complex nanoparticles, termed metal-complex multi-wall nano-capsules, were fabricated with a lipid core (sorbitan monostearate dispersed in oil), polysorbate 80 and lecithin as surfactant stabilizers, and an outer wall of chitosan with zinc ion complexation, and had ~150-nm diameter sizes. Lipid NCs without the chitosan-zinc coating were also tested in parallel, and the DHA-containing metal-complex multi-wall NCs were additionally functionalized with antibody targeting elements in some cases. DHA-containing nanoparticles with antibody functionalization were efficiently up-taken by endothelial cells, as compared to other DHA-containing nanoparticles, which supports the feasibility of developing this nano-carrier potentially further for anti-atherosclerosis applications. Specific formulations were also identified that did not affect endothelial cell viability, which could be particularly advantageous.

While polyunsaturated fatty acids such as DHA and EPA have been widely explored in nano-carrier formulations for anti-inflammatory activities, there is outstanding potential to further investigate other classes of biologically active fatty acids and monoglycerides for immunomodulatory applications. For example, GML has been reported to be a promising T cell-suppressive agent that could help to treat autoimmune diseases [90].

### 3.4. Drug Delivery

While free fatty acids and monoglycerides have long been explored as permeation enhancers to facilitate intestinal absorption of pharmaceutical drugs [91], there is also evidence that fatty acids encapsulated within nanostructured assemblies can increase membrane permeability and facilitate more effective drug delivery [92]. An early example of this concept demonstrated that DHA and EPA enrichment of phosphatidylcholine liposomes enhanced permeability of, and transport through, cell monolayers while there was negligible change in cell viability [93]. Such permeability enhancing properties of DHA-encapsulated liposomes are also useful for modulating the membrane fluidity of neuronal cells, which can help to support neuroprotective effects, for example [90,94].

By utilizing a polymer-oil nanostructured carrier design [95], Guo et al. developed ~100-nm diameter, transferrin-functionalized nanoparticles that contained 5–20% DHA along with an antiretroviral drug, darunavir, to facilitate blood–brain barrier (BBB) passage and potential treatment of viral infections in the brain [96]. With increasing DHA content in the nanoparticles, darunavir release was slowed down during the initial stage but more extensive over time, which can be advantageous to avoid challenges with initial burst release. The DHA-containing nanoparticles also had low cytotoxicity against BBB endothelial cells in vitro and cellular uptake of darunavir was enhanced for nanoparticle formulations containing 15% DHA, as compared to those with 5% DHA and free DHA. In addition, the darunavir-containing nanoparticles had greater antiviral activity against human immunodeficiency virus (HIV) than free darunavir. Intravenous administration of the nanoparticles in mice also improved darunavir accumulation in the brain. These findings demonstrate the potential utility scope of lipid nanoparticles for brain delivery applications, especially when functionalized with targeting ligands.

## 4. Conclusions and Outlook

The aforementioned examples demonstrate the potential of lipid nanoparticle technologies to deliver biologically active fatty acids and monoglycerides. In certain fields such as antibacterial and anticancer therapies, there is a growing number of academic studies, while curiously the range of biologically active fatty acids and monoglycerides that has been tested remains relatively narrow. It would be advantageous to expand this range and to also consider which applications might yield the most promising translational outcomes in terms of regulatory landscape and needs. Below, we critically discuss selected themes within this scope and emphasize forward-looking topics that can help to bridge the gap between the current status of the field and real-world applications.

### 4.1. Engineering Optimization

While fatty acids and monoglycerides have often been discussed interchangeably as lipophilic, membrane-interacting compounds that disturb pathogenic membranes, recent biosensing advances such as the cell-membrane-mimicking, supported lipid bilayer (SLB) platform [97] have revealed new insights into how different fatty acids and monoglycerides interact with membrane structures. For example, Thid et al. demonstrated that DHA, at bulk concentrations above its critical micelle concentration (CMC) value, can induce tubule formation in zwitterionic SLBs [98]. On the other hand, the anionic character of DHA affects its membrane-interacting properties with negatively charged SLBs [99]. These findings provided initial clues into how biologically active fatty acids interact with lipid bilayers and also how such measurement tools can guide the mechanistic evaluation of membrane interactions in different environmental conditions.

More recently, these SLB capabilities have been extended to characterize medium-chain fatty acids and monoglycerides. Using this approach, Yoon et al. demonstrated that LA fatty acid and GML monoglyceride induce tubule and budding formation, respectively [100]. These distinct interaction profiles were attributed to the anionic and nonionic properties of LA and GML, respectively, and corresponding effects on membrane translocation and membrane strain induction. Early work focused on phospholipid SLBs while a later study showed that LA and GML can also trigger the same pattern of membrane remodeling behavior in phospholipid/cholesterol SLBs [101]. It was further revealed that CA fatty acid and GMC monoglyceride exhibit a similar trend in membrane-interaction behaviors and the compounds are mainly active in the micellar state, with appreciably lower levels of membrane disruption in the monomeric state [102]. The potency of fatty acids can be tuned by the solution pH because this condition affects their charge state and hence CMC values, while monoglycerides are nonionic and exhibit consistent CMC values and stable membrane-interaction behaviors across standard pH conditions [103]. Ether-linked analogues of monoglycerides, which also have pH-stable molecular properties, have also demonstrated promising membrane-disruptive activities in related studies [104,105].

Together, these results emphasize the importance of expanding the scope of fatty acids and monoglycerides that are utilized in lipid nanoparticles since each type has unique membrane-interaction properties. Perhaps the most promising direction involves exploring combinations of different fatty acids and monoglycerides to achieve enhanced biological functions. For example, GML/LA mixtures can induce distinct membrane morphological changes and greater degrees of membrane disruption compared to GML or LA alone [106]. Combinations of different monoglycerides can also modulate the degree of membrane budding [107]. Interestingly, however, such studies have only been conducted with free compounds and future efforts should be expanded to tracking the interactions of lipid nanoparticles with SLB platforms and membrane models. The need for such studies is further enhanced by emerging applications, e.g., antiviral mitigation in feed and drinking water [108,109], that would benefit from dilution-stable formulations of biologically active fatty acids, monoglycerides, and combinations thereof.

There is also excellent potential to utilize pathogen-membrane-mimicking SLB platforms and there has been much progress in developing bacterial cell membrane models [110,111]. It is envisioned that these biosensing technologies can serve as a predictive model to evaluate membrane-interacting activities of fatty acids and monoglycerides in free form and within various types of nanostructured assemblies.

### 4.2. Ocular Applications

Currently explored applications of lipid nanoparticles incorporating biologically active fatty acids and monoglycerides are focused on skin and systemic infections, while another key opportunity would be the utilization of biologically active lipids to treat ocular infections. The eye is an organ that is inaccessible from other systemic sites due to blood-aqueous, blood-retinal, and blood-vitreous barriers [112]. Therefore, topical ophthalmic delivery is the most frequent way to deliver drugs to the eye.

One key target is *Neisseria gonorrhoeae* (*N. gonorrhoeae*), which can cause corneal scarring, eye perforation, and blindness [113]. To date, researchers have screened a wide range of fatty acids and fatty acid derivatives and identified that GMC monoglyceride and myristoleic fatty acid are promising compounds to prophylactically inhibit *N. gonorrhoeae* infection [114]. It has been further demonstrated that there is a particularly high barrier for *N. gonorrhoeae* to develop resistance against GMC, while myristoleic acid is more susceptible to resistance development [115]. An eye drop formulation, which was composed of 0.25% MC, 1% hydroxypropyl methylcellulose, and 1% polysorbate 20, has also been developed and shown to kill *N. gonorrhoeae* bacteria in vitro and ex vivo [116]. Surfactant-containing microemulsions that incorporate LLA fatty acid are also being developed and have been shown to inhibit *N. gonorrhoeae* and *S. aureus* [117], while it would be advantageous to further explore other lipid-nanoparticle-based delivery platforms such as cationic nano-emulsions [118] and surfactant-free emulsions that could have improved performance and biocompatibility.

Indeed, the eye is a complicated organ with sophisticated dynamic (reflex blinking, tear secretion, dose spillage, and nasolacrimal drainage) and static (poor permeability of corneal epithelium) barriers that significantly limit ophthalmic product residence on the eye surface, restrict drug absorption, and obstruct the treatment of ocular diseases, including infections [119]. Cationic oil-in-water NEs are promising systems for topical ophthalmic drug delivery due to increased residence time on the eye surface, which is attributed to electrostatic interactions of positively charged oil nanodroplets with negatively charged ocular surface mucins [112]. The oil nanodroplets gradually break down over time and the released oil molecules can subsequently intercalate into the lipid layer of the tear film whereas the surfactant molecules can merge with the muco-aqueous gel layer [120,121]. Within this context, the oil nanodroplets can act as a reservoir of lipophilic drugs, such as fatty acids and monoglycerides, that can be delivered to the lipid layer of the tear film. Subsequent fusion of NE oil droplets with bacterial cell membranes could potentially occur and result in releasing the biologically active components to destabilize the pathogen [122].

Considering the membrane-disruptive properties of fatty acids and monoglycerides, a crucial aspect of ophthalmic NE development in this case is the investigation of its effect on the corneal epithelial barrier. The safe concentration range of formulation components and candidate formulations has to be evaluated in vitro by employing 3D corneal models. The immortalized human corneal epithelial cell line (HCE-T) is the most extensively characterized human-derived cell line and therefore the most frequently used in corneal 3D models [123,124]. In order to better mimic dynamic eye barriers and therefore more reliably predict nanoparticle safety, it is advised to use models that provide dynamic flow conditions such as the recently developed DynaMiTES model [125,126]. As such, there is enormous potential to develop lipid nanoparticle technologies for ocular applications in future efforts.

### 4.3. Translational Opportunities

Until now, most application examples have focused on liposomal formulations of fatty acids that were developed in proof-of-concept efforts, without systematic investigation of how the loading of free fatty acids affects the structure, functionality, and biocompatibility of the liposomal structures. Such strategies have shown high potential for topical application and local eradication of pathogenic bacteria in the gastrointestinal tract [19]. Nowadays, SLNs, NLCs, and lipid NCs are emerging as perhaps even better carriers of fatty acids and especially monoglycerides, in which case these molecules can simultaneously act as the building blocks and biologically active compounds [53,127,128]. However, the structural features of these lipid nanoparticles such as size and surface properties, loading capacity, preparation method suitable for large-scale production, efficiency, and safety still need to be investigated and optimized [122]. There also needs to be a more rigorous focus on safety, including in vitro and in vivo cytotoxicity evaluation and dosing considerations—a topic which is covered in some reports in vitro, as discussed above, but most reports focus on therapeutic performance only. The mechanisms of selectivity for pathogenic membranes in various contexts, e.g., viral membranes, bacterial cell membranes, cancer cell membranes, over normal cell membranes must also be better understood. A related aspect, especially for intravenously administered lipid nanoparticles, is complement activation-related pseudo-allergy (CARPA), which is an adverse immune response that can be predicted in vitro and in porcine models in vivo [129]. Furthermore, co-encapsulation of antimicrobial drugs into nanocarriers built from fatty acids and monoglycerides provides endless possibilities for the development of advanced formulations that are able to simultaneously deliver multiple therapeutics and, at the same time, modulate drug-pathogen interactions to perhaps overcome antibiotic resistance [19].

As an example of the translational prospects that lie ahead for lipid nanoparticles and the broader potential of carrier strategies to deliver biologically active fatty acids and monoglycerides in different contexts, cyclodextrins are emerging as another potential carrier that can solubilize and stabilize fatty acids and monoglycerides through inclusion complex formation for various molecular biology applications [130,131,132]. Cyclodextrin complexation provides a suitable technology platform to overcome the unfavorable characteristics of fatty acids and monoglycerides by converting semisolid or liquid compounds into more technologically feasible compactable powders with enhanced chemical stability and more acceptable sensory characteristics [133,134]. For example, cyclodextrin complexes of OA and LA are commercially available as supplements for cell culture media, where methylated β-cyclodextrin (MeβCD) supports the solubility, chemical stability, and delivery of fatty acids in culture media. Compared to albumin, MeβCD can transfer its entire fatty acid load and is less likely to extract cell nutrients or introduce impurities. Moreover, dilutions in media can be filtered and are stable during storage for months [135]. A recent study indicated that cyclodextrin-mediated lipid exchange may provide a new method to characterize the phospholipids in the outer leaflet of red blood cells, providing a tool to study erythrocyte biology and modulate cell–macrophage interactions [136].

Cyclodextrins can also potentially improve transport of fatty acids and monoglycerides into bacterial biofilms [137] and reduce local tissue irritation [138,139], providing the possibility of safely applying higher lipid doses and offering exciting application opportunities, which should be explored also with lipid nanoparticles. Cyclodextrin-fatty acid inclusion complexes can also be used as a building block to develop novel nanoparticle carriers with advanced functionalities [140]. Finally, through careful selection of the cyclodextrin type, it might be possible to further enhance the biological activity of included lipids, as observed with the inflammatory responses of human monocyte cells to n-3 polyunsaturated fatty acids [141]. It should also be noted that some cyclodextrin derivatives alone have antibacterial activity and act as pore-forming agents, showing the potential for synergistic activity with antimicrobial lipids [142]. Moreover, cholesterol extraction by MeβCD can cause the disruption of lipid rafts that block downstream processes related to host cell entry of membrane-enveloped viruses and consequently can impair the infectivity of such viruses [143,144]. The synergistic effect of cyclodextrins and membrane-disruptive disinfectants against several enveloped viruses and bacteria was recently demonstrated [145], emphasizing the possibility that cyclodextrin might also enhance the antiviral activity of fatty acids and monoglycerides. Rather than view lipid nanoparticles and cyclodextrins as competing technologies, the relatively advanced regulatory status and application opportunities demonstrated by cyclodextrins highlight the potential that lies ahead for lipid nanoparticle technology, especially when considering the supramolecular organizational features and membrane-mimicking environments that lipid nanoparticles can offer, and the different carriers might have particular advantages in specific contexts.

### 4.4. Regulatory Considerations

While the development of innovative (nano)carriers of biologically active fatty acids and monoglycerides is highly attractive for academic investigation, their potential for transfer to everyday clinical practice must also consider issues such as system complexity, difficult scalability, and potential challenges related to large-scale production according to Good Manufacturing Practice (GMP) standards, high material and manufacturing costs, batch-to-batch reproducibility, limited storage stability, and potential toxicity. During the scale-up of laboratory methods, sometimes the desired features of nanoparticles, such as particle size, drug encapsulation efficiency, process residual materials, colloidal stability, surface morphology, and even therapeutic outcomes, may change [146]. For example, increases in impeller speed and agitation time during nanoparticle preparation by the emulsion method may result in a decrease in particle size and consequently lower cargo entrapment efficiency [147]. Furthermore, the bottom-up approach, often used during laboratory scale production of nanoparticles, is less popular at the industrial level as it requires the removal of remaining solvent traces, which is a complicated and costly process. Moreover, the lipid film hydration method, which is the most commonly used method for liposome production at the laboratory level, is not applicable for their preparation at an industrial scale, in which case ethanol injection is most often used [27]. In general, large-scale manufacturing of nanoparticulate drug carriers is a long and laborious process characterized by the number of unit operations and associated tests that are quite exhaustive, imposing many difficulties in complying with GMP requirements. Very often, new technologies, like microfluidic devices [148] and other technological inventions [149], should be introduced into the production process, which typically requires significant investment. The scale-up and large-scale production of nanoparticles become even more complicated and expensive when sterility of the final product is required [150]. All of these issues must be considered when translating lipid nanoparticle technologies into commercial products.

Finally, the global regulatory framework for nanoparticle drugs is still being developed, especially when it comes to structural components doubly serving as biologically active components and building blocks [151]. In this light, conventional formulations should not be neglected and a focus on simple nanoparticle design should be prioritized to facilitate translation when possible. For example, palmitoleic acid (1% *w/w*) showed significant antibacterial properties against *S. aureus* in vitro when formulated as a surfactant-free, oil-in water emulsion with triolein (1–3% *w/w*) and water [152]. Another promising example is the antibacterial and anti-biofilm activity against Gram-positive and Gram-negative pathogens of a non-aqueous 5% (*w/w*) GML monoglyceride gel that was observed in vitro [153]. However, this particular formulation did not succeed in clinical trials, in which case the GML gel was observed to be no more clinically or microbiologically effective than a placebo in curing bacterial vaginitis and caused an unexpected increase of *Lactobacillus sp.* count in the treated group [154]. While such efforts are promising in the sense that biologically active fatty acids and monoglycerides are reaching human clinical trials, the results also highlight probably the biggest obstacle in the translation of fatty acids and monoglycerides to everyday clinical practice–the outstanding need to develop more relevant and predictive in vitro models for preclinical assessment and validation of the activity and safety of developed (nano)formulations [16].

### 4.5. Closing Remarks

While there remain numerous challenges in translating current scientific insights into clinically ready lipid nanoparticle medicines involving fatty acids and monoglycerides, there is room to be optimistic in the face of exciting research advances over the past decade. For example, the recent impact of rapidly scaled up lipid nanoparticle technologies for vaccine delivery applications offer an inspiring vision. Many such challenges described above were also cited when developing lipid nanoparticles for vaccine delivery and these challenges were overcome in the face of a global health challenge and through interdisciplinary collaboration. Considering the prevalent threat of membrane-enveloped viruses and bacteria along with the continuing rise of cancer and inflammatory diseases worldwide, we anticipate that ongoing developments in the lipid nanoparticle technology field will lead to unprecedented capabilities for delivering biologically active fatty acids and monoglycerides to treat medical diseases and improve human health.

## Figures and Tables

**Table 1:** Summary of antibacterial application examples utilizing lipid nanoparticles that incorporate biologically active fatty acids and monoglycerides ^1,2^.

Fatty Acid/ Monoglyceride	Bacteria (In Vitro/ In Vivo)	Formulation (Size)	Key Effects	Ref.
LA	*P. acnes* (+) In vitro	Liposomes composed of Egg PC ^3^, cholesterol, and ~0–69 mol% LA (~120-nm diameter)	- LipoLA was more potent against *P. acnes* than free LA in vitro, provided there is a critical amount of LA per liposome. - LipoLA fuses with *P. acnes* bacterial membrane to release LA into bacterial cell membrane.	[42]
LA	*P. acnes* (+) In vivo	Liposomes composed of Egg PC ^3^, cholesterol, and ~0–69 mol% LA (~120-nm diameter)	- LipoLA showed bactericidal effects against *P. acnes* in mouse ear model, applied via intradermal injection or topically. - LipoLA did not cause skin irritation commonly caused by acne medications.	[43]
LA	*P. acnes* (+) In vitro, in vivo	Liposomal gel formulation co-loaded with curcumin and LA	- In vitro, curcumin-LA liposomal gel was more potent against *P. acnes* than curcumin-only gel. - In vivo, curcumin-LA liposomal gel induced greater *P. acnes* bacterial load reduction than curcumin-only gel in mouse ear model.	[44]
OA	*S. aureus* (+) In vitro, in vivo	Liposomes composed of Egg PC ^3^, cholesterol, and ~0–61 mol% OA (~80-nm diameter)	- LipoOA was more potent against methicillin-resistant *S. aureus* than free OA in vitro. - LipoOA had significantly greater bactericidal effects compared to bare liposomes in vivo on mouse skin.	[45]
LLA	*H. pylori* (-) In vitro	Liposomes composed of Egg PC ^3^, cholesterol, and ~61 mol% LLA (~88-nm diameter)	- LipoLLA killed both spiral and coccoid forms of *H. pylori*. - LipoLLA treatment had no resistance development while LLA triggered resistant strains to emerge.	[46]
LLA, OA, SA	*H. pylori* (-) In vitro	Liposomes composed of Egg PC ^3^, cholesterol, and ~50 mol% SA, OA, or LLA (~85-nm diameters)	- LipoLLA was the most potent, followed by LipoOA, while LipoSA showed no antibacterial effect. - LipoLLA caused greater membrane disruption than LipoOA.	[47]
LLA	*H. pylori* (-) In vivo	Liposomes composed of Egg PC ^3^, cholesterol, and 51 mol% LLA (~106-nm diameter)	- LipoLLA was more potent against *H. pylori* than free LLA in vitro. - In *H. pylori*-infected mouse stomach, LipoLLA reduced *H. pylori* bacterial load significantly compared to conventional antibiotic treatment.	[48]
LLA	*H. pylori* (-) In vivo	Liposomes composed of Egg PC ^3^, cholesterol, and 51 mol% LLA (~110-nm diameter)	- In *H. pylori*-infected mouse stomach, LipoLLA caused less gastrointestinal side effects compared to conventional antibiotic treatment.	[49]
GML	*S. aureus* (+) In vitro	Bicelles composed of DOPC ^4^ loaded with ~29–95 mol% GML (~100–600-nm diameters)	- DOPC/GML bicelles showed membrane-disruptive activity that was distinct from that of free GML, and bicelles with ~80 mol% GML caused pore formation. - Bicelles with ~80 mol% GML were bactericidal against *S. aureus* at 0.125 mM effective GML concentration.	[50]
LA	*P. acnes* (+) In vitro	Polymeric micelles composed of PCL-PEG-PCL ^5^ loaded with ~89–96 mol% LA (~30–90 nm diameters)	- LA-loaded micelles were more potent against *P. acnes* than free LA in vitro. - Efficacy of LA-loaded micelles increased with the amount of loaded LA.	[51]
SA, LA, OA	*P. aeruginosa* (-) In vitro	SLNs loaded with 32 mol% SA + 68 mol% LA; 45 mol% SA + 49 mol% LA + 6% OA; or 35 mol% SA + 59 mol% LA + 6% OA (~150–190 nm diameters)	- All SLN formulations were bactericidal, while the addition of OA enhanced SLN antimicrobial activity. - The SLN formulations could reduce bacterial adhesion on surfaces by ~99%.	[52]
COA, CYA, CA, LA, MA, PA, SA, GMC, GML	*S. aureus* (+), *P. aeruginosa* (-), *A. baumannii* (-) In vitro	LNCs ^6^ loaded with ~20 mol% of various fatty acids (~40–90 nm diameters)	- All fatty acid-LNCs (FA-LNCs) and monoglyceride-LNCs (MG-LNCs), but not LNCs without FA or MG, inhibited Gram-positive *S. aureus*, while Gram-negative bacteria were more resistant. - MG-LNCs were more potent than FA-LNCs against bacteria. - Longer chain length in antimicrobial lipids increased activity against Gram-positive bacteria and decreased activity against Gram-negative bacteria.	[53]
GML	*S. aureus* (+) In vitro	LNCs 6 ^6^ co-loaded with ~21 mol% GML and antimicrobial peptides (AMPs) (~40-nm diameter)	- Methicillin-susceptible *S. aureus* was more sensitive to GML-LNCs than methicillin-resistant *S. aureus*. - GML-LNCs exerted time-dependent bactericidal effects on *S. aureus* strains. - AMPs and GML-LNCs acted in synergy against *S. aureus*, i.e., their combination was more potent than GML-LNCs or AMPs alone.	[54]
GML	*S. aureus* (+) In vivo	LNCs ^6^ co-loaded with ~21 mol% GML and AMPs (~30–130 nm diameter)	- GML-LNCs showed bactericidal activity against four different *S. aureus* strains. - GML-LNCs significantly reduced bioluminescent *S. aureus* bacterial count in an infected mouse wound-healing model.	[55]
DHA	*H. pylori* (-) In vitro	NLCs ^7^ loaded with ~15–30 mol% DHA (~300-nm diameter)	- DHA-loaded NLCs were more potent against *H. pylori* than free DHA or unloaded NLCs. - DHA-loaded NLCs act on H. *pylori* by causing membrane fragmentation and leakage of cytoplasmic contents.	[56]

^1^ Fatty acid and monoglyceride abbreviations: caproic acid (COA), caprylic acid (CYA), capric acid (CA), docosahexaenoic acid (DHA), lauric acid (LA), myristic acid (MA), palmitic acid (PA), stearic acid (SA), linolenic acid (LLA), glycerol monocaprate (GMC), and glycerol monolaurate (GML); ^2^ Liposomal formulations of LA, OA, SA, and LLA are denoted by LipoLA, LipoOA, LipoSA, and LipoLLA, respectively. ^3^ Egg PC = egg phosphatidylcholine; ^4^ DOPC = 1,2-dioleoyl-*sn*-glycero-3-phosphocholine; ^5^ PCL-PEG-PCL = poly(ε-caprolactone)-poly(ethylene glycol)-poly(ε-caprolactone); ^6^ LNC was composed of poly-oxyl 15 hydroxy-stearate surfactant, lecithin, caprylic/capric acid triglycerides, and fatty acid or monoglyceride as a co-surfactant. ^7^ NLC was composed of a mixture of glyceryl palmitostearate, medium-chain triglycerides, Tween-60 surfactant, and DHA.

**Table 2:** Summary of anticancer application examples utilizing lipid nanoparticles that incorporate biologically active fatty acids and monoglycerides ^1^.

Fatty Acid/ Monoglyceride	Cancer Cell Line (In Vitro/In Vivo)	Formulation (Size)	Key Effects	Ref.
OA, LNA, ALA, EPA, DHA, and ethyl esters	Lung carcinoma, colon tumor, stomach tumor cells In vitro	HLs composed of DMPC ^2^, Tween 80, and ~0–100 μg/mL polyunsaturated fatty acid (PUFA) (~50–200 nm diameters)	- Over 1 month, HL-PUFAs remained stable in size, while blank HLs had aggregation-related size increases. - HL-PUFA inhibited cancer cells more potently than those of control liposomes.	[66]
LNA, DHA	Lung carcinoma, colon tumor, stomach tumor cells In vitro	HLs composed of DMPC ^2^, Tween 80, and ~0–200 μg/mL LNA or DHA (<210-nm diameters)	- HL-LNA inhibited tumor cell growth through necrosis-inducing mechanisms that involved lipid peroxidation. - HL-DHA inhibited tumor cell growth through apoptosis-inducing mechanisms that did not depend on lipid peroxidation.	[67]
DHA	Stomach tumor cells In vitro	Liposomes composed of DMPC ^2^ and 10–60 mol% DHA (~100 nm diameter)	- DMPC/DHA liposomes inhibited cancer cell growth more potently than DMPC liposomes.	[68]
DHA	Colon carcinoma cells In vivo	Liposomes composed of DMPC ^2^ and 50 mol% DHA (~100-nm diameter)	- DMPC/DHA liposomes reduced number of metastatic nodules and induced greater apoptosis in metastatic tumor mouse model than DMPC liposomes. - Longer survival time was observed in mouse model after treatment with DMPC/DHA liposomes than with DMPC liposomes.	[69]
DHA	Breast cancer cells In vitro	Liposomes composed of ether and phytanyl PC lipids ^3^ and 10 mol% DHA (~140-nm diameter)	- Liposomal DHA was stable in the pH range of 1 to 7.4 and less prone to oxidation than free DHA over 1–2 weeks. - Liposomal DHA was more effective than free DHA in inhibiting cancer cell growth and malignant transformation.	[70]
OA	Breast and lung cancer cells In vitro	Liposomes composed of DPPC ^4^ phospholipids, cholesterol, and 40 mol% DHA (~130-nm diameter)	- OA-loaded liposomes were cytotoxic to tumor cells while bare liposomes did not inhibit the tumor cells.	[71]
DHA	Breast cancer cells In vitro	NLC ^5^ loaded with doxorubicin (DOX) and 20 mol% or 40 mol% DHA (~80-nm diameter)	- NLC-DHA/DOX was more cytotoxic than free DOX/DHA for both cancer cells. - NLC-DHA/DOX had higher drug uptake in both cancer cells and penetration in tumor spheroid models than free DHA/DOX.	[72]
DHA	Breast cancer cells In vivo	NLC ^5^ loaded with DOX and ~20 mol% DHA (~70-nm diameter)	- NLC-DHA/DOX treatment significantly inhibited tumor growth compared to blank-NLC or NLC-DOX treatment.	[73]
DHA	Breast cancer cells In vitro, in vivo	NLC ^5^ loaded with DOX, α-tocopherol succinate (TS), and ~20 mol% DHA (~80-nm diameter)	- NLC-DHA/DOX/TS had higher antitumor activity in vitro than NLC-DOX and NLC-TS. - In vivo, NLC-DHA/DOX/TS showed the highest tumor growth inhibition, compared to NLC-DOX and blank NLC.	[74]
DHA	Breast cancer cells In vitro	PEG-PLA NCs; NEs; and NLCs ^6^, all co-loaded artemether (ART) and DHA triglycerides (~150–200 nm diameters)	- All 3 lipid nanocarriers improved ART/DHA selectivity and cytotoxicity towards tumor cells, compared to free-ART/DHA or blank-nanocarriers. - NEs had the highest cellular uptake compared to NCs and NLCs.	[75]
DHA	Lung cancer cells In vitro	SLNs ^7^ loaded with DOX and ~0–4 mol% DHA (~100-nm diameter)	- SLN-DHA/DOX was 3 times more effective against cancer cells than free DHA/DOX. - SLN-DHA/DOX had much higher cellular uptake and DOX accumulation than free DHA/DOX or blank SLN.	[76]
DHA, LNA	Colon carcinoma cells In vitro	RV-based SLN ^8^ loaded with ~0.1 mol% DHA or LNA (~840-nm and ~1000-nm diameters for SLN-DHA and SLN-LNA, respectively)	- SLN encapsulation significantly increased LNA and DHA in cancer cells, compared to free LNA and free DHA. - SLN-DHA and SLN-LNA greatly inhibited cancer cell growth and proliferation compared to free DHA and free LNA.	[77]

^1^ Fatty acid abbreviations: oleic acid (OA), linoleic acid (LNA), α-linolenic acid (ALA), eicosapentaenoic acid (EPA), and docosahexaenoic acid (DHA); ^2^ HL and DMPC stand for hybrid liposome and l-α-dimyristoylphosphatidylcholine, respectively. ^3^ 1,2-di-*O*-hexadecyl-*sn*-glycero-3-phosphatidylcholine and 1,2-di-*O*-phytanyl-*sn*-glycero-3- phosphatidylethanolamine ether lipids were used. ^4^ DPPC = 1,2-dipalmitoyl-sn-*glycero*-3-phosphocholine. ^5^ NLC oily phase comprised Tween 80, oleic acid, doxorubicin, triethanolamine, glyceryl di-behenate, and DHA; aqueous phase comprised EDTA and water. ^6^ Both NEs and NLCs had poloxamer 188 as the aqueous phase within the nanoparticle design. ^7^ SLN oily phase comprised Tween 80, butylated hydroxytoluene, doxorubicin, triethanolamine or stearyl-amine, and DHA; aqueous phase comprised EDTA and water. ^8^ SLN matrix was composed of R3,5,4′-trihydroxy-trans-stilbene, also known as resveratrol (RV).
